# The HicA toxin from *Burkholderia pseudomallei* has a role in persister cell formation

**DOI:** 10.1042/BJ20140073

**Published:** 2014-03-28

**Authors:** Aaron Butt, Victoria A. Higman, Christopher Williams, Matthew P. Crump, Claudia M. Hemsley, Nicholas Harmer, Richard W. Titball

**Affiliations:** *Biosciences, College of Life and Environmental Sciences, University of Exeter, Geoffrey Pope Building, Stocker Road, Exeter EX4 4QD, U.K.; †School of Chemistry, University of Bristol, Cantock's Close, Bristol BS8 1TS, U.K.

**Keywords:** *Burkholderia pseudomallei*, HicAB, melioidosis, persister cell, toxin–antitoxin, CFU, colony-forming units, dsRBD-like, dsRNA-binding domain-like, MIC, minimum inhibitory concentration, TA, toxin–antitoxin, TBST, TBS supplemented with 1% (v/v) Tween-20

## Abstract

TA (toxin–antitoxin) systems are widely distributed amongst bacteria and are associated with the formation of antibiotic tolerant (persister) cells that may have involvement in chronic and recurrent disease. We show that overexpression of the *Burkholderia pseudomallei* HicA toxin causes growth arrest and increases the number of persister cells tolerant to ciprofloxacin or ceftazidime. Furthermore, our data show that persistence towards ciprofloxacin or ceftazidime can be differentially modulated depending on the level of induction of HicA expression. Deleting the *hicAB* locus from *B. pseudomallei* K96243 significantly reduced persister cell frequencies following exposure to ciprofloxacin, but not ceftazidime. The structure of HicA(H24A) was solved by NMR and forms a dsRBD-like (dsRNA-binding domain-like) fold, composed of a triple-stranded β-sheet, with two helices packed against one face. The surface of the protein is highly positively charged indicative of an RNA-binding protein and His^24^ and Gly^22^ were functionality important residues. This is the first study demonstrating a role for the HicAB system in bacterial persistence and the first structure of a HicA protein that has been experimentally characterized.

## Abstract 2

*B. pseudomallei* HicA toxin plays a role in differentially regulating bacterial persister cell frequencies after exposure to ceftazidime or ciprofloxacin. The molecular structure of HicA toxin revealed an RNA-binding-like fold. His^24^ and Gly^22^ are important for HicA function.

## INTRODUCTION

*Burkholderia pseudomallei* is a motile Gram-negative bacterium that normally resides in soil in South-East Asia and northern Australia [1]. It is an opportunistic human pathogen, entering the host by inhalation, skin abrasions or ingestion and causing the disease melioidosis [2]. In spite of antibiotic intervention, melioidosis has a mortality rate of up to 44% in endemic areas, and it is the third most frequent cause of death from infectious diseases in north-east Thailand [3]. Up to 15% of patients relapse following completion of therapy [4]. The bacterium is considered to be a potential bioterrorism agent, partly owing to its ability to infect by the airborne route [1]. Little is known about the mechanisms by which this bacterium establishes disease or the mechanisms that allow it to establish infections that are refractory to antibiotic treatment [2,5].

Persister cells were first identified in the 1940s. *Staphylococcus aureus* cultures treated with supra-lethal doses of antibiotic showed biphasic killing, with a subpopulation of bacterial cells surviving the treatment [6]. When these antibiotic-tolerant bacteria were grown in fresh media and exposed to antibiotic, they generated similar frequencies of survivors, showing they were not a genetically defined subpopulation. Instead, tolerance appeared to be due to phenotypic variation within the bacterial population. More recently, it has been suggested that persister cells act as a reservoir for chronic infections [7,8], and it has been shown that a broad range of bacterial species can form persister cells. *Escherichia coli* can form persister cells at a frequency of between 10^−6^ and 10^−5^ in the exponential phase and up to 1% in the stationary phase [9,10], whereas our data indicate that *B. pseudomallei* can form persister cells at frequencies of up to 10^−1^ (C. M. Hemsley, J. X. Luo, C. Andreae, C. Butler, O. S. Soyer and R. W. Titball, unpublished work). A current model suggests that persister cells are generated as a consequence of fluctuations in the expression levels of certain genes and that this noise can be amplified by external signals [11]. Indeed, a series of quorum sensing-like chemicals such as indole, CSP (competence signalling protein) and pyocynanin have been associated with the formation of *E. coli*, *Streptococcus mutans* and *Pseudomonas aeruginosa* persister cells [12–14].

The genetic mechanisms that underpin persister cell formation are poorly defined, but there is evidence that TA (toxin–antitoxin) modules play a role. Transcriptome profiling of *E. coli* and *Mycobacterium tuberculosis* persister cells has revealed differential expression of TA systems [10,15]. In *E. coli*, exposure to ciprofloxacin increased TisB toxin levels and, in parallel, increased persister cell numbers [16]. However, the most direct evidence for a role of TA modules in persister cell formation is derived from studies showing that mutations in the HipBA TA system of *E. coli* can modulate the frequency of persister cell formation [17–19]. Similarly, in *M. tuberculosis* it has been reported that inactivation of the RelE genes influences the frequency of persister cell formation [20].

We identified recently four candidate TA systems in *B. pseudomallei* [21]. TA systems can be categorized into five groups (type I, II, III, IV or V) based on the gene product [22–25]. Typically, one gene encodes a 95–135-amino-acid toxin. These toxins can interact with cellular components such as RNA, ribosomes or DNA gyrase, resulting in a bactericidal or bacteriostatic response within the cell [23]. The cognate antitoxin (RNA or protein) binds to the toxin and blocks its activity [26]. The toxin and antitoxin show strong inter-dependence, with the antitoxin usually being indispensable.

One of the TA systems we identified in *B. pseudomallei* was homologous to the *E. coli* HicBA system. The *E. coli* HicA toxin induces cleavage of mRNA and tmRNA (transfer-messenger RNA), thereby preventing translation [27]. The *E. coli hicAB* locus is transcribed in response to amino acid and carbon starvation and HicA activity is dependent on the Lon protease. This protease degrades the unstable HicB antitoxin, which acts as an auto-repressor of the locus, subsequently allowing transcription of the genes in response to starvation [27]. In *E. coli*, the *hicBA* system has been shown to play a role in growth, with overexpression of the toxin resulting in bacteriostasis [27]. Expression of *hicB* was able to neutralize *hicA*-induced bacteriostasis. We have shown similar phenotypes when *B. pseudomallei* K96243 HicA or HicA and HicB were expressed in *E. coli* MG1655 [21]. However, little is known about the biological roles of HicBA.

The aim of the present study was to investigate the biological role(s) of the *B. pseudomallei hicAB* system and to establish how the structure of the toxin is related to these roles. We show through overexpression and deletion of *hicA* a role for this toxin in the generation of persister cells. Using mutagenesis and structure analysis, we also reveal residues that are critical for the biological activity of HicA and suggest a function for the toxin in RNA degradation. The present study is the first to demonstrate a role for the HicAB system in bacterial persistence and the first structure of a HicA toxin that has been experimentally validated.

## EXPERIMENTAL

### Bacterial strains, growth conditions and chemicals

Bacterial strains used in the present study are listed in Table 1. Unless otherwise stated, bacteria were grown in LB broth at 37°C with shaking (200 rev./min) or on LB agar plates at 37°C. Where appropriate, the medium was supplemented with 100 μg/ml ampicillin (Sigma–Aldrich) for selection of pBAD/His (Invitrogen), 15 μg/ml tetracycline for selection of pME6032, 50 μg/ml or 300 μg/ml trimethoprim for selection of pSCrhaB3, 50 μg/ml kanamycin for selection of pET26-b and 25 μg/ml chloramphenicol for maintenance of the Rosetta plasmid. Where indicated, glucose, arabinose or rhamnose were added to a final concentration of 0.2% or IPTG was added to a final concentration of 25 mM or 0.5 mM. Autoinduction media and labelled media were prepared as described previously [28].

**Table 1:** Bacterial strains used or created in the present study

Bacterial strain	Genotype/comments	Source
*E. coli* K-12 MG1655	F^−^ λ^−^ ilvG-rfb-50 rph-1	Laboratory strain collection
*E. coli* DH5α λ*pir*	ΔlacU169(ΦlacZΔM15), recA1, endA1, hsdR17, thi-1, gyrA96, relA1, λpir phage lysogen	Laboratory strain collection
*E. coli* S17-1 λpir	TpR SmR recA, thi, pro, hsdR-M^+^RP4:2-Tc:Mu:Km Tn7 λpir	Laboratory strain collection
*E. coli* DH5α (pRK2013)	ΔlacU169(ΦlacZΔM15), recA1, endA1, hsdR17, thi-1, gyrA96, relA1, pRK2013 (KmR oriColE1 RK2-Mob^+^ RK2-Tra^+^)	Laboratory strain collection
*E. coli* Rosetta(DE3)pLysS	F^−^ ompT hsdS_B_(R_B_^−^ m_B_^−^) gal dcm λ(DE3 [lacI lacUV5-T7 gene 1 ind1 sam7 nin5]) pLysSRARE (Cam^R^)	Novagen
*E. coli* XL-10 Gold	endA1 glnV44 recA1 thi-1 gyrA96 relA1 lac Hte Δ(mcrA)183 Δ(mcrCB-hsdSMR-mrr)173 tet^R^ F’[proAB lacI^q^ZΔM15 Tn10(Tet^R^ Amy Cm^R^)]	Stratagene
*B. pseudomallei* K96243	Clinical isolate	Laboratory strain collection
*B. pseudomallei* K96243 *ΔhicAB*	K96243 derivative. Unmarked deletion *ΔBPSS0390-0391*	The present study

### Construction of plasmids

The *hicA* and *hicB* genes were PCR amplified from *B. pseudomallei* K96243 genomic DNA by Hotstart taq polymerase (Qiagen). *hicA* was amplified using primers hicA_forward (5′-GGAGCTCGCCATGGCTATGAACTCATC-GAAGCTGATCC-3′) and hicA_reverse (5′-GAATTCTCACA-GGCCGGCGGATTTC-3′) and cloned into NcoI- and EcoRI-digested pBAD/His (Invitrogen) to create pBAD-*hicA* or primers hicAtag_forward (5′-CGCCGAGCTCATGAAC-TCATCGAAGCTGATCC-3′) and hicA_reverse and cloned into SacI- and EcoRI-digested pBAD/His to create pBAD/His-*hicA*. pSCrhaB3/His-*hicA* was created by digesting pBAD/His-*hicA* with NcoI and HindIII and the isolated *hicA* cassette was cloned into the corresponding sites of pSCrhaB3 [29]. pET26-b/His-*hicA* (H24A) was made by digesting pBAD/His-*hicA* (H24A) with NcoI and EcoR1 and the isolated *hicA* (H24A) cassette was cloned into the respective sites of pET26-b (Novagen). The *hicB* gene was amplified using primers hicB_forward (5′-GAGCTCATGGAATTTCCCATCGCAGTG-3′) and hicB_reverse (5′-CCATGGTTATGCGTGCCTAACTTTGCC-3′) and cloned into NcoI- and SacI-digested pME6032 [30].

### Construction of the *B. pseudomallei* Δ*hicAB* mutant

PCR was used to amplify both 600 bp upstream of the HicAB locus using primers hicAB_LF_1 (5′-ATAT-AACCCGGGTCTCGTGCTGACCGGCCC-3′) and hicAB_LF_2 (5′-CGATATTTAGCGGCCGCCATTAGCTCCCCCGAA-TGTC-3′) and 600 bp downstream using hicAB_RF_1 (5′-GA-GCTAATGGCGGCCGCTAAATATCGCGTGCGCCTGTA-3′) and hicAB_RF_2 (5′-GAGCTCATGGTCGCTGGATTGGGTGT-3′). The PCR product also contained regions of sequence that were specific for the start and end of the *hicAB* locus in order for homologous recombination to occur. The upstream and downstream PCR products were used as template DNA for a second recombinant PCR using hicAB_LF_1 and hicAB_RF_2 primers to create the knockout cassette, which was cloned into pDM4 [29] via the XmaI and SacI restriction sites. The resulting plasmid was transformed into *E. coli* S17λ and then conjugated into *B. pseudomallei* K96243 to create a merodiploid strain. Colonies were plated on to LB agar supplemented with 10% (w/v) sucrose to drive *sacB* expression in pDM4 and promote recombination. Δ*hicAB* mutants were confirmed by PCR and sequencing of the resulting DNA fragment.

### Co-expression assay

Co-expression was performed as described previously [21]. Briefly, early exponential phase cultures of *E. coli* pBAD/His-*hicA* (or mutant allele)/pME6032-hicB were supplemented with 0.2% arabinose and IPTG to induce expression of *hicA* and *hicB* respectively for 2 h. The CFU (colony-forming units) fold change was calculated as the difference between CFU observed after 2 h of expression, compared with that observed before expression.

### Persister cell assay

For *E. coli* MG1655/pBAO-*hicA*/pME6032-*hicB*, overnight cultures were diluted 1:100 in 30 ml of fresh LB supplemented with antibiotic for plasmid selection and grown at 37°C and shaking at 200 rev./min until the *D*_590_ of the culture reached approximately 0.1. Cultures were supplemented with either 0.2% glucose or 0.002–0.2% arabinose to repress or induce expression of the toxin. These cultures were incubated for 3 h at 37°C, 200 rev./min. After standardizing to 2×10^8^/ml cells in LB broth, 500 μl of culture was mixed with 500 μl of 200×MIC (minimum inhibitory concentration) of antibiotic in a 24-well plate. Plates were incubated for up to 30 h at 37°C. At the indicated time points, cultures were washed twice in LB broth. Samples were serially diluted and plated on to LB plates supplemented with ampicillin, tetracycline and 1 mM IPTG to induce antitoxin expression from the pME6032 vector. Persister frequency was calculated as CFU at 24 h divided by CFU at *T*_0_. *B. pseudomallei* K96243 ∆*hicAB*/pSCrhaB3-*hicA* was treated as above, except that cultures were supplemented with 0.2% glucose or 0.2% rhamnose to repress or induce expression of the toxin. These cultures were incubated for 4 h at 37°C, 200 rev./min before treating with antibiotic. Cultures were plated on to LB agar supplemented with trimethoprim and 0.2% glucose. For *B. pseudomallei* K96243 and *B. pseudomallei* K96243 ∆*hicAB*, ~10^8^ (*D*_590_=0.1) stationary or early exponential phase cells were incubated with 100×MIC ciprofloxacin or ceftazidime for the indicated time in a 24-well plate. Cells were washed in LB broth and plated on to LB agar for enumeration.

### Site-directed mutagenesis

Site-directed mutagenesis was carried out on pBAD/His-*hicA* toxin DNA using the QuikChange® lightning kit (Agilent), following the manufacturer's instructions. Plasmid DNA containing the mutation was transformed into XL10-gold ultracompetent cells. Mutations were confirmed by sequencing.

### Large-scale protein expression

Overnight cultures of *E. coli* Rosetta(DE3) harbouring pET26-b constructs were grown in a 5 ml volume of LB supplemented with antibiotic. Cultures were diluted 1:100 in four 100 ml aliquots of ZYM-5052 autoinduction medium [28] supplemented with 50 μg/ml kanamycin and 25 μg/ml chloramphenicol. Cultures were incubated at 300 rev./min, 37°C until reaching mid-exponential phase. The temperature was then reduced to 20°C and growth continued overnight. For ^13^C,^15^N-labelling, overnight cultures were grown in LB broth before diluting 1:100 in 2 litres of fresh LB. These cultures were grown to a *D*_590_ of approximately 0.8–0.9 before harvesting the cultures at 3000 ***g*** for 10 min in sterile 250 ml tubes to collect the cell pellets. The cell pellets were then washed in PBS before resuspending in 500 ml [^13^C,^15^N]N-5052 medium (replacing all carbon sources with 4 g/l [^13^C]glucose) [28]. The cultures were incubated at 37°C for a further 1 h and 0.5 mM IPTG was then added to induce expression of the cloned *hicA*(H24A) gene. The temperature was reduced to 20°C for maximal expression and grown overnight.

### Protein extraction

Bacterial cultures were harvested by centrifugation at 12000 ***g*** for 10 min. The supernatant was then discarded. The cell pellet was resuspended in lysis mixture [10 μl of benzonase, 10 μl of lysozyme and 10 ml of Bugbuster (Novagen) per 1 g of cell pellet] by vortex-mixing and then incubated on a rocker for 30 min until the cell mixture appeared translucent. Following lysis, the cell debris was harvested by centrifugation at 16000 ***g*** for 20 min. The supernatant was collected for subsequent purification.

To check for expression of histidine-tagged proteins, 1 ml of cell culture at a cell density of *D*_590_ of 1.0 was harvested by centrifugation for 2 min at 17000 ***g***. Cell pellets were resuspended in a lysis mixture (100 μl of Bugbuster, 1 μl of benzonase and 1 μl of lysozyme), incubated and then centrifuged for 1 min at 17000 ***g*** to separate the soluble and insoluble material.

### Protein purification

Cell lysate containing histidine-tagged protein was purified using nickel-affinity-based column systems (His GraviTrap, GE Healthcare) following the manufacturer's protocol. The protein was de-salted by running through a PD-10 desalting column (GE Healthcare). The PD-10 columns were first equilibrated with 25 ml TBS (20 mM Tris/HCl, pH 7.4, and 150 mM NaCl) or PBS (20 mM phosphate, pH 7.4, and 150 mM NaCl). The histidine tag was removed by incubating the protein with enterokinase (New England Biolabs) for 16 h at 23°C. A Superdex 75 16/60 HR column (GE Healthcare) was used for size-exclusion gel chromatography to exclude any impurities from the protein preparation. The protein was concentrated using an Amicon Ultra-15 Centrifugal Filter Unit with Ultracel 3-kDa membrane by centrifugation at 3200 ***g***, 4°C. Protein concentration was determined using a Nanodrop 1000 (Thermo Scientific). An extinction coefficient of 0.863 at λ=280 nm was used for the calculation as determined using Biology workbench [30].

### Protein detection

Samples were boiled at 95°C for 5 min in loading dye before electrophoresis on Novex SDS/PAGE (4–12% gels) (Invitrogen). Following separation, the protein was stained using Simply Blue (Invitrogen). Protein was blotted on to a nitrocellulose membrane using an iBlot dry blotting system (Invitrogen) for 5–7 min at 20–23 V. The membrane was stored at 4°C overnight in TBST [TBS supplemented with 1% (v/v) Tween-20] containing 3% (w/v) BSA for blocking. It was then incubated with TBST plus 3% (w/v) BSA containing anti-xpress epitope antibody (Invitrogen) diluted 1:5000 and incubated for 1 h at room temperature (19°C). The membrane was washed three times in TBST before adding secondary horseradish peroxidase-conjugated goat anti-mouse antibody [1:10000 dilution in TBST plus 3% (w/v) BSA]. The wash steps were repeated as described above. The protein was detected using SuperSignal West Femto Substrate (Thermo Scientific) and chemiluminescence on a Chemidoc imager equipped with QuantityOne software (Bio-Rad Laboratories).

### NMR spectroscopy

The concentration of the final NMR sample was typically 1 mM dissolved in PBS (20 mM phosphate and 150 mM NaCl) with 10% (v/v) ^2^H_2_O. All NMR experiments were acquired at 25°C on a cryoprobe-equipped Varian VNMRS 600 MHz spectrometer. The backbone resonances were assigned using HNCA and HNCO spectra and side-chain resonances using 3D ^15^N-edited TOCSY, HCCH-TOCSY and ^13^C-edited NOESY spectra. Distance restraints for structure calculations were derived from aliphatic and aromatic ^13^C-edited and ^15^N-edited NOESY spectra as well as a 2D ^1^H-^1^H NOESY spectrum recorded at 20°C. All of the NMR data were processed using NMRPipe [31], and the spectra were visualized and assigned using CcpNmr Analysis version 2.2 [32]. Structure calculations were performed using Aria 2.3 [33] using spin diffusion correction throughout, network anchoring in the first three iterations, and 8000 cooling steps in both the cool1 and cool2 phases. Structures (100) were calculated in iteration 8, of which the ten lowest energy structures were further water refined. PSVS 1.4 [34] and CING [35] were used to validate the structures and NMR data. The chemical shifts and structural restraints were deposited with the Biological Magnetic Resonance Data Bank (BMRB) [36] under accession number 19464. The structure was deposited with the PDB under code 4C26.

## RESULTS AND DISCUSSION

### *B. pseudomallei* forms antibiotic-tolerant persister cells

First, we investigated persister cell formation in *B. pseudomallei* K96243 using ceftazidime and ciprofloxacin. Ceftazidime is routinely used in front-line treatment of melioidosis [2], whereas the use of ciprofloxacin to treat melioidosis is currently being investigated [37].

**Figure 1:**
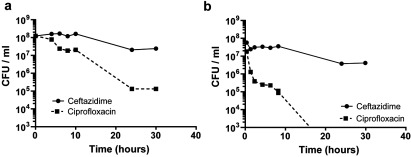
Killing of *B. pseudomallei* with 100×MIC (200 μg/ml) ciprofloxacin or ceftazidime over 30 h (**a**) Stationary phase cultures (*D*_590_=3.0), (**b**) exponential phase (*D*_590_=0.2). The data are shown as the means±S.E.M. for at least two biological repeats.

Exponential or stationary phase *B. pseudomallei* cultures were standardized to a *D*_590_ of 0.1, incubated with either 100×MIC (200 μg/ml) of ceftazidime or ciprofloxacin and the number of culturable cells determined over 30 h by plating on to LB agar (Figure 1). Killing of stationary phase *B. pseudomallei* by either antibiotic occurred over the first 24 h (Figure 1a). Further incubation did not result in further cell death, but revealed a population of cells that could tolerate the antibiotic. This subpopulation equated to approximately 10^−3^ (0.1%) and 10^−1^ (10%) of the total bacterial population for ciprofloxacin and ceftazidime respectively. Our finding that cefatzidime, a beta-lactam antibiotic, was active against stationary phase cultures is in accordance with the definition of the stationary growth phase of bacterial cultures, where there may be balanced growth and death of *B. pseudomallei* cells [38].

When exponential phase *B. pseudomallei* were exposed to ceftazidime, approximately 5% of the population survived after 30 h (Figure 1b). When cultures were treated with ciprofloxacin, cell numbers dropped by approximately 250-fold after 8 h and were below detectable levels of <10^3^ CFU/ml [<10^−5^ (0.001%) survivor frequency] after 24 h (Figure 1b).

After treatment of either the exponential or stationary phase cultures with antibiotic, we checked that the subpopulation of surviving bacteria were not spontaneous antibiotic-resistant mutants. Colonies were re-streaked in parallel on to LB agar or LB agar containing 10×MIC ciprofloxacin or ceftazidime respectively. Bacterial growth only occurred on LB agar and not on LB agar plus antibiotic (results not shown).

Overall, our findings that the treatment of *B. pseudomallei* cultures with otherwise supra-lethal doses of antibiotic resulted in biphasic killing, and that survivors were not spontaneous antibiotic-resistant mutants, confirms the presence of persister cells [39–41]. Our finding that the frequency of *B. pseudomallei* persister cells was lower in exponential phase cultures than in stationary phase cultures is similar to the results reported in *E. coli* [42]. These differences in frequencies of persisters revealed after exposure to ciprofloxacin or ceftazidime suggests the presence of different persister populations in *B. pseudomallei*.

### *hicA* overexpression causes growth inhibition in *B. pseudomallei* K96243 *ΔhicAB*

We have shown previously that expression of the *B. pseudomallei* HicA toxin in *E. coli* resulted in the rapid cessation of bacterial growth and a reduction in the number of culturable cells [21]. These phenotypes were reversible on co-expression of the cognate HicB antitoxin [21], confirming that HicA acts bacteriostatically. We next investigated whether overexpression of HicA in *B. pseudomallei* K96243 resulted in growth inhibition. The *B. pseudomallei hicA* gene was cloned into the broad host range plasmid pSCrhaB3, and the plasmid was conjugated into *B. pseudomallei* K96243 wild-type.

Cultures of wild-type *B. pseudomallei* harbouring pSCrhaB3-*hicA* grew similarly when glucose or rhamnose was added to repress or induce expression (results not shown).

This lack of toxicity of *hicA* in wild-type *B. pseudomallei* K96243 may be due to the expression of the endogenous chromosomally encoded HicB sequestering the toxin. Other groups have also reported the lack of toxic activity after expressing toxins in the wild-type host of other bacterial species [43,44]. To test this possibility, we constructed a *B. pseudomallei* K96243 mutant lacking the *hicAB* locus (*ΔhicAB*) and transformed the HicA-expressing plasmid (pSCrhaB3-*hicA*) into this strain.

**Figure 2:**
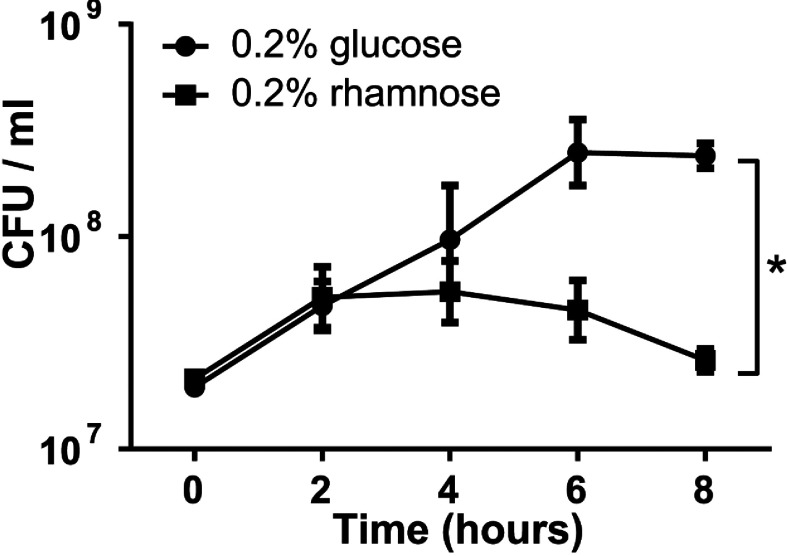
Culturability of a *ΔhicAB* mutant of *B. pseudomallei* containing the plasmid cloned *hicA* gene Expression of *hicA* was induced with 0.2% rhamnose or repressed with 0.2% glucose. At the times indicted, samples were plated on to LB agar and colonies enumerated. The data are shown as the means±S.E.M. for three biological repeats. *P<0.05, following an unpaired Student's *t* test as determined using the Holm–Sidak method for multiple comparisons.

When treated with glucose the number of culturable bacteria increased ~20-fold over the next 8 h (Figure 2). In contrast, when rhamnose was added to induce *hicA* expression, the number of culturable bacteria increased for the first 2 h (~5-fold), but then declined to levels significantly lower than the control (Figure 2). Control *B. pseudomallei* K96243*ΔhicAB* harbouring empty pSCrhaB3 plasmid grew similarly in medium containing glucose or rhamnose (results not shown) confirming that these results were a consequence of the specific induction of *hicA* expression.

Although we observed the cessation of growth when *hicA* was expressed in the *ΔhicAB* mutant of *B. pseudomallei*, we did not see the loss of culturability (i.e. a decline in CFU) that we reported previously when *hicA* was expressed in *E. coli* [21]. This might reflect a number of possible differences between the *B. pseudomallei* and *E. coli* hosts including differences in the levels of expression of *hicA*, different targets of HicA, variation in the stability of the proteins or the ability of other antitoxins present in these host cells to partially neutralize the activity of HicA.

### Overexpression of *hicA* in *E. coli* reduces bacterial killing by ceftazidime or ciprofloxacin

We next investigated the effect of *B. pseudomallei* HicA on *E. coli* persister cell formation by exploiting the ability of HicA to render cells non-culturable and the ability of HicB to rescue the culturability of cells [21]. In this assay, *hicA* expression was induced, the cells exposed to antibiotic and then plated on to agar containing IPTG to induce *hicB* expression and restore culturability [21]. This way, we could measure whether the dormancy induced by *hicA* expression had provided resistance to antibiotics. For these studies, we used *E. coli* MG1655 encoding the *hicA* toxin cloned into pBAD/His and the *hicB* antitoxin cloned into pME6032 (i.e. *E. coli* containing *hicA* and *hicB* on different plasmids and expressed from different promoters). Western blotting of an *E. coli* MG1655 pBAD/His *hicA/*pME6032-*hicB* cell lysate following arabinose induction confirmed promoter-driven *hicA* expression. Conversely, under glucose repression, no detectable band was present (results not shown).

**Figure 3:**
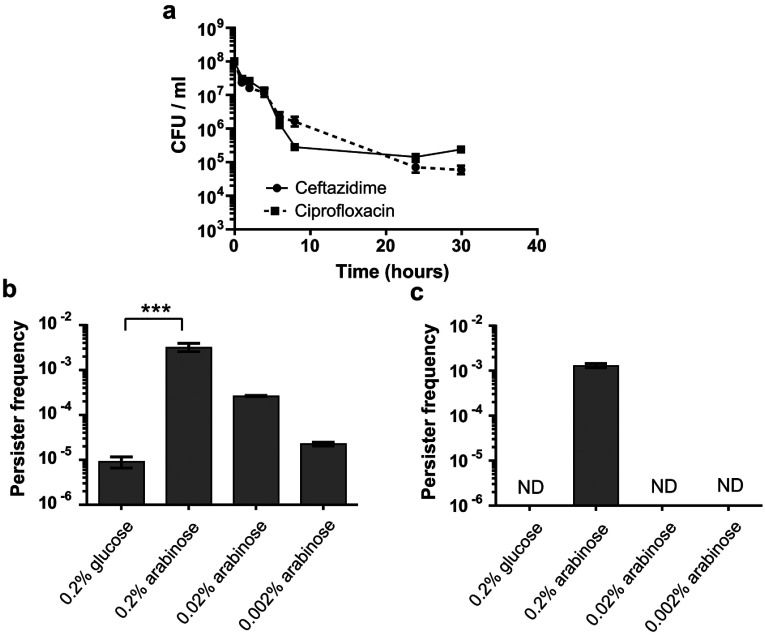
Persister cell formation in *E. coli* MG1655 encoding the *hicA* toxin cloned into pBAD/His and the *hicB* antitoxin cloned into pME6032 Standardized exponential phase cultures were incubated with arabinose or glucose to induce or repress *hicA* expression respectively, before treating with antibiotic. (**a**) *hicA* was induced with 0.2% arabinose for 3 h and then incubated with 100×MIC ciprofloxacin or ceftazidime and CFU tracked over 30 h. (**b**) *hicA* was repressed with 0.2% glucose and induced with a range of arabinose concentrations (0.002–0.2%) for 3 h before treatment with 100×MIC ciprofloxacin for 24 h. (**c**) As described for (**b**) except treatment was with 100×MIC ceftazidime. In both (**b**) and (**c**), persister frequencies were calculated as CFU numbers post-antibiotic treatment divided by CFU numbers pre-antibiotic treatment. The data are shown as the means±S.E.M. for three biological repeats. ***P<0.001, as determined using one-way ANOVA with Tukey's post test. ND, no detectable colonies.

Subsequently, this strain was grown to early exponential phase and *hicA* expression was induced with 0.2% arabinose for 3 h. Killing by 100×MIC ciprofloxacin or ceftazidime (32 μg/ml and 20 μg/ml respectively) was tracked over 30 h (Figure 3a). For cultures incubated with ceftazidime, there was bacterial killing for the first 8 h, but further incubation revealed a surviving antibiotic tolerant population at a frequency of approximately 10^−3^ (0.1%). A similar frequency of drug-tolerant persister cells was observed following ciprofloxacin treatment after 24 h. Re-streaking antibiotic-treated cultures on to LB agar or LB agar plus antibiotic revealed growth on LB agar, but not on LB agar containing antibiotic (results not shown), confirming that the survivors were not antibiotic-resistant mutants.

The induction of *hicA* expression increased the frequency of bacteria surviving ciprofloxacin exposure, in an arabinose concentration-dependent manner, from approximately 10^−5^ (under *hicA* repressed conditions) to 10^−3^ (0.1%) of the bacterial population at the highest arabinose concentration (Figure 3b). The frequency of ceftazidime survivors increased from less than 10^−6^ (under *hicA* repressed conditions) to 10^−3^ when *hicA* expression was induced with 0.2% arabinose, but no increase in survi-vors was seen at lower arabinose concentrations (Figure 3c). An *E. coli* control strain harbouring empty vectors showed no alteration in persister frequency when supplemented with either sugar (results not shown). Therefore overexpression of *B. pseudomallei hicA* in *E. coli* increased ciprofloxacin and ceftazidime persister frequencies. Furthermore, the differences in the dependency of persister frequencies to antibiotics with different mode of actions on HicA expression levels suggests that ceftazidime and ciprofloxacin persisters might represent different populations that are generated by a different mechanism.

### Deletion of *hicAB* or overexpression of *hicA* in *B. pseudomallei* differentially modifies ceftazidime or ciprofloxacin persister cell frequencies

**Figure 4:**
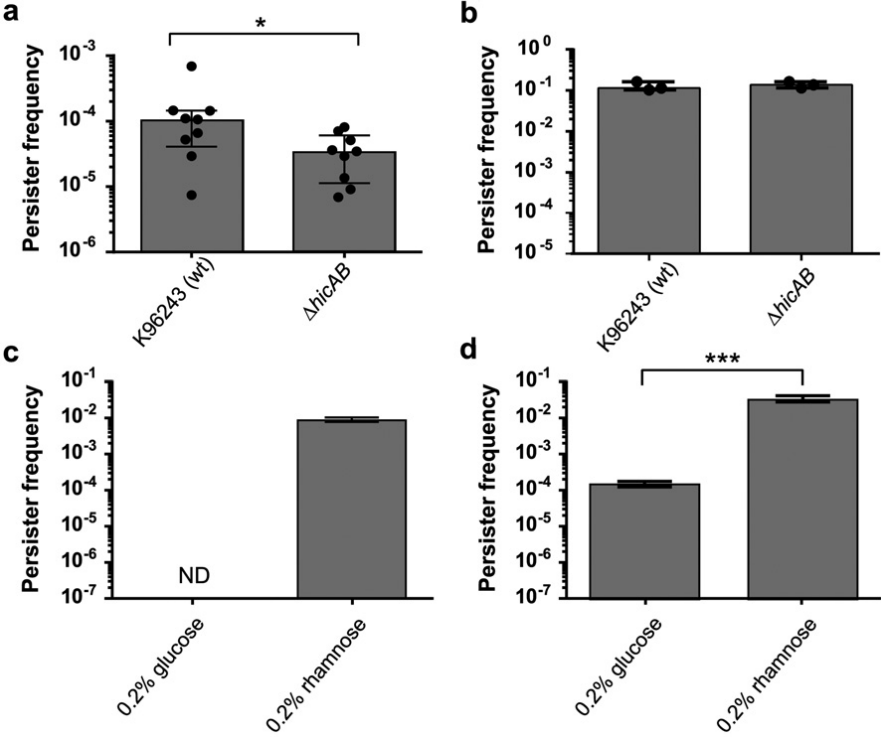
Effect of deletion or overexpression of *hicA* on persister cell formation in *B. pseudomallei* K96243 (**a** and **b**) Stationary phase *B. pseudomallei* K96243 or *B. pseudomallei* K96243 Δ*hicAB* treated with 100×MIC (200 μg/ml) ciprofloxacin (**a**) or ceftazidime (**b**) respectively for 24 h. The data are shown as the means±S.E.M for at least three biological repeats. *P<0.05, as determined using a Wilcoxon matched-pairs signed rank test. (**c** and **d**) Cultures of *B. pseudomallei* K96243 Δ*hicAB* with pSCrhaB3 cloned *hicA* were grown to early exponential phase before repressing or inducing expression of *hicA* with 0.2% glucose or 0.2% rhamnose for 4 h. Standardized cultures were then exposed to 100×MIC ciprofloxacin (**c**) or ceftazidime (**d**) respectively for 24 h. Persister frequencies were calculated as CFU numbers post-antibiotic treatment divided by CFU numbers pre-antibiotic treatment. The data are shown as the means±S.E.M. for at least three biological repeats. ***P<0.001, as determined a Student's *t* test. ND, no detectable colonies; wt, wild-type.

We next investigated the effect of deletion of the *hicAB* locus in *B. pseudomallei*. When *B. pseudomallei* K96243 or *B. pseudomallei* K96243 Δ*hicAB* early stationary phase samples were exposed to 100×MIC of ciprofloxacin for 24 h, the ∆*hicAB* mutant had reduced persister frequencies of 7-fold compared with the wild-type (*P*<0.05, Wilcoxon matched-pairs signed rank test; Figure 4a). In contrast, when these strains were exposed to 100×MIC ceftazidime for 24 h, there was no difference in persister cell frequencies (Figure 4b).

In spite of the evidence linking TA systems with persistence, the deletion of individual TA modules rarely results in a reduction in persister cell frequencies. This has been attributed to redundancy between TA systems [45] and the deletion of multiple TA systems was needed to elicit a significant reduction in persister cell frequency in *E. coli* MG1655 [46]. However, in *M. tuberculosis* a change in persister frequency has been reported when individual *relE* genes were inactivated [20]. Also, deletion of the *mqsR* toxin gene or *mqsRA* TA pair in *E. coli* BW52133 reduced persister frequencies following ampicillin treatment by 6- and 7-fold respectively [47]. Our finding that deletion of *hicBA* in *B. pseudomallei* only affects the survival towards one class of antibiotic also suggests that ceftazidime and ciprofloxacin persisters are different subpopulations.

Next, *hicA* was overexpressed in *B. pseudomallei* ∆*hicAB.* The plasmid cloned *hicA* gene was repressed or expressed after the addition of 0.2% glucose or 0.2% rhamnose for 4 h and standardized cultures were then incubated with 100×MIC ciprofloxacin or 100×MIC ceftazidime. When *hicA* expression was repressed, the number of culturable cells following ciprofloxacin treatment was below the limit of detection (<100 CFU), indicating a persister frequency of <10^−7^ (Figure 4c). When *hicA* expression was induced with rhamnose, the frequency of persister cells increased significantly (*P*<0.001, as determined using a Student's *t* test) to approximately 10^−2^. Similarly, after exposure of cultures to ceftazidime the frequency of culturable cells increased significantly (*P*<0.001, as determined using a Student's *t* test) from approximately 10^−4^ in *hicA*-repressed cultures to 10^−1^ in *hicA*-induced cultures (Figure 4d). *B. pseudomallei* ∆*hicAB* harbouring empty pSCrhaB3 showed no difference in survival following exposure to 100×MIC ciprofloxacin or 100×MIC ceftazidime when grown in medium containing either 0.2% glucose or 0.2% rhamnose (results not shown).

Our findings that toxin overexpression increased persister frequencies are similar to the results reported by other workers. For instance, expression of RelE in *E. coli* increased the number of persisters to cefotaxime, ofloxacin or tobramycin by 10–10000-fold [48], whereas overexpression of HipA increased persisters by 10000-fold [19]. Overexpression of RelE in *M. tuberculosis* increased persister frequencies up to 13-fold [20]. In contrast with *E. coli*, HicB expression was not required to re-awaken or reveal *B. pseudomallei* persisters in our experiments. Our results likely reflect the greater potency of the HicA toxin when expressed in *E. coli*. More broadly, our results are similar to those reported by Keren et al. [48] where RelB expression was not needed to reveal persisters in a RelE-induced *E. coli ∆relBE* strain.

One of the key findings from the present study is the differential role of HicA in the frequency of persister cells towards two different antibiotics. We showed that the low level expression of *hicA* in *E. coli* resulted in an increase in ciprofloxacin persister cells, but high level expression of *hicA* was necessary for an increase in ceftazidime persister cells. Similarly, the deletion of *hicAB* in *B. pseudomallei* reduced the number of ciprofloxacin persisters, but did not affect the numbers of bacteria surviving ceftazidime treatment. Previously, Singh et al. [20] reported that three RelE homologues in *M. tuberculosis* had different roles in promoting survival towards different antibiotics. It was suggested that this reflected differences in the RNA target specificity of these toxins. It is possible that our findings reflect the differential cleavage of mRNA which was dependent on the concentration of HicA. Overall, our findings support an increasing understanding of the complexity of the relationship between toxin expression and survival towards different antibiotics.

### His^24^ and Gly^22^ are important for HicA toxicity

**Figure 5:**
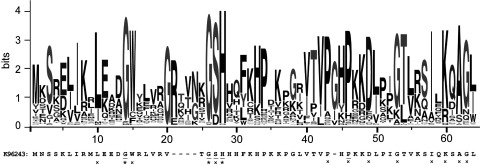
Graphical representation of the amino acid residues in 75 homologous HicA sequences following sequence alignment The amino acid sequence of *B. pseudomallei* K96243 HicA. The asterisks (*) and crosses (×) indicate >90% and >80% amino acid conservation respectively. Residues that were chosen for mutagenesis are underlined. Sequences were aligned using Clustal Omega [47] and the graphic generated using Web logo [48].

**Figure 6:**
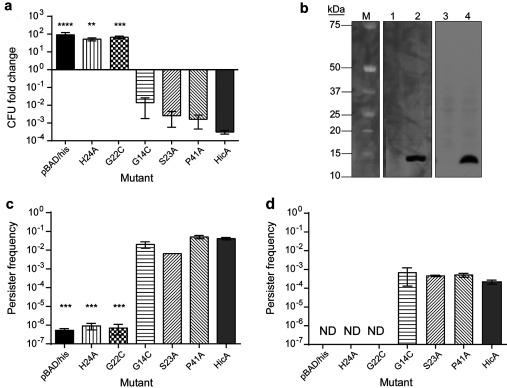
Functional analysis of site-directed *hicA* mutants (**a**) Fold change in the number of culturable *E. coli* MG1655 cells harbouring pBAD/His or pBAD/His encoding wild-type *hicA* or mutant alleles. Cultures were grown to early exponential phase before inducing expression of *hicA* or mutants with 0.2% arabinose for 2 h. The data are shown as the means±S.E.M. for three biological repeats. **P<0.01, ***P<0.001 and ****P<0.0001, as determined using one-way ANOVA and Dunnett's post-test, comparing the means with HicA. (**b**) Western blot of *E. coli*-expressing H24A (lanes 1 and 2) or G22C (lanes 3 and 4) HicA variants under glucose-repressed (lanes 1 or 3) or arabinose-induced (lane 2 and 4) conditions. The reactive band size is 13 kDa. The molecular mass (M) is indicated in kDa on the left-hand side. (**c** and **d**) Persister cell formation by *E. coli* MG1655 expressing wild-type HicA or HicA mutants after 100×MIC antibiotic treatment. Cultures were grown to early exponential phase before inducing expression of the toxin or toxin mutants. Standardized cultures were incubated with 100×MIC of ciprofloxacin (**c**) or ceftazidime (**d**) for 24 h in a 24-well plate at 37°C. After washing, cells were plated on to LB agar. Persister frequency was calculated as CFU counts post antibiotic treatment divided by CFU counts pre-treatment. The data are shown as the means±S.E.M. for three biological repeats. ***P<0.001, as determined using a one-way ANOVA with a Dunett's post-test, comparing the means with HicA. ND, no detectable colonies.

To identify potential catalytic residues, the sequence of HicA was aligned with 75 other sequences of proteins identified as potential homologues (*E* value <0.0001) (Supplementary Figure S1 at http://www.biochemj.org/bj/459/bj4590333add.htm). In total, 14 residues were at least 80% conserved (Figure 5). The histidine residue at position 24 in HicA was most highly conserved. Using site-directed mutagenesis, His^24^, Gly^22^, Gly^14^, Ser^23^ and Pro^41^ were individually changed to alanine or cysteine. The toxic activity of the proteins was assessed by measuring the change in the number of culturable bacteria following expression of the mutant alleles in *E. coli* MG1655. Cultures of *E. coli* expressing the G14C, S23A or P41A mutants showed a decrease in the number of culturable cells, which was comparable with *E. coli* expressing wild-type *hicA* (Figure 6a). In contrast, *E. coli* expressing the G22C or H24A mutants grew similarly to control *E. coli* cells harbouring the empty pBAD/His plasmid. The difference between the viable counts of *E. coli* expressing G22C or H24A compared with *E. coli* expressing wild-type HicA was significant (*P*<0.01 or *P*<0.001, as determined using a one-way ANOVA Dunnett's post-test) and expression of the non-toxic alleles was confirmed by Western blotting. In summary, we have identified two residues, Gly^22^ and His^24^, which play a role in HicA toxicity.

### Gly^14^, Ser^23^ and Pro^41^ do not have a role in antitoxin binding

Even though the G14C, S23A and P41A mutations had no effect on toxicity, the residues might have been important for antitoxin binding. Therefore all three mutants were tested in a co-expression assay in *E. coli*, where the wild-type or mutant toxin alleles were cloned into pBAD/His and the *hicB* antitoxin gene was cloned into pME6032. After induction of both the TA genes, culturable bacteria were enumerated after plating on to agar. The number of culturable bacteria was similar for *E. coli* expressing wild-type HicA or the G14C, S23A or P41A variants (results not shown). Expression of the mutant alleles was confirmed by Western blotting (results not shown).

### His^24^ and Gly^22^ are important in the persister phenotype

Finally, the mutant toxin alleles were tested for their abilities to modulate persister cell frequencies. *E. coli* strains harbouring individual site-directed mutants of the HicA toxin (cloned into pBAD/His) and pME6032-*hicB* were grown to early exponential phase. Expression of the toxin genes was induced with 0.2% arabinose for 3 h. Cultures were then standardized and incubated with 100×MIC of ciprofloxacin or 100×MIC of ceftazidime for 24 h. *E. coli* expressing the toxic G14C, S23A or P41A alleles had persister frequencies similar to the positive *hicA* control strain following ciprofloxacin or ceftazidime treatment (Figure 6c and 6d respectively). In contrast, expression of the non-toxic H24A and G22C alleles in *E. coli* resulted in persister frequencies similar to the empty vector control following ciprofloxacin or ceftazidime treatment and significantly different to frequencies in *E. coli* expressing *hicA* (*P*<0.001, as determined using a one-way ANOVA Dunnett's post-test). In summary, only the two residues that are required for HicA-mediated bacteriostatis, Gly^22^ and His^24^, also play a role in inducing persister cell formation.

### The structure of HicA forms a dsRBD-like (dsRNA-binding domain-like) fold

The identification of a stable non-toxic variant form of HicA allowed us to overexpress the protein and determine the structure using NMR. Previously, information on the likely structure of HicA toxins has been derived from the reported structure of TTHA1913, a protein of unknown function from *Thermus thermophilus*, which on the basis of a sequence alignment belongs to the HicA family [49].

**Figure 7:**
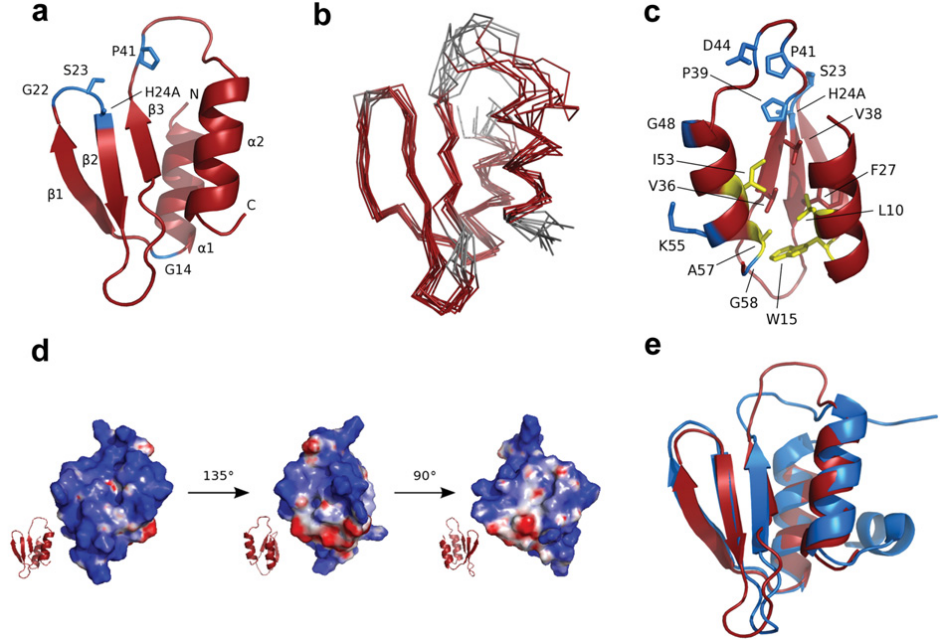
Structure analysis of HicA (**a**) Structure of the HicA(H24A) protein with the positions of the five residues that were selected for mutagenesis coloured blue. The N- and C-termini and secondary structure elements are indicated. (**b**) Ensemble of ten calculated structures of HicA(H24A). Disordered residues (as calculated by PSVS 1.4 [34]) are coloured grey. (**c**) Structure of HicA(H24A) with side chains of conserved residues shown as sticks (blue and yellow); yellow residues contribute to the hydrophobic core, as do Val^36^ and Val^38^ and Phe^27^, indicated as red sticks. Residues 6–2 have been omitted from all panels, as they are completely unstructured. (**d**) Charge distribution of the surface of HicA(H24A). Regions of positive, negative and no charge are coloured blue, red and white respectively. (**e**) Superimposition of HicA(H24A) (red) with TTHA1913 from *T. thermophilus* (blue; PDB code 1WHZ).

The *hicA*(H24A) mutant gene expressed in pBAD/His was not toxic to *E. coli* and therefore the HicA(H24A) protein could be purified in sufficient quantities for structure determination by NMR (Figure 7). ^13^C,^15^N-double-labelled HicA(H24A) was prepared in order to conduct triple-resonance experiments for resonance assignment and restraint generation. Unfortunately, the ^13^C-labelling was not uniform; backbone carbonyl atoms as well as certain side-chain carbon atoms (e.g. valine, leucine, isoleucine and histidine C_β_, and proline C_δ_) did not appear to be labelled above the natural abundance level. It is currently unclear where the natural abundance carbon source stems from; however, repeated expression gave the same result and further studies are required to investigate whether HicA(H24A) is having other unforeseen effects on *E. coli* metabolism. Resonance assignment was thus hampered by the fact that HNCO-based experiments could not be used and numerous side-chain sites lack assignments (Supplementary Table S1 at http://www.biochemj.org/bj/459/bj4590333add.htm). It was nonetheless possible to derive sufficient numbers of NOEs to calculate a high-quality structure of HicA(H24A) (Supplementary Table S1). As predicted previously [49], HicA toxin forms a dsRBD-like fold consisting of a triple-stranded β-sheet with two α-helices packed against one of its faces (Figures 7a and 7b). The long loop connecting the β3 strand with the α2 helix is ill-defined due to a lack of NOEs, suggesting that this loop occupies multiple conformations in solution. The hydrophobic core is formed by Leu^10^, Trp^15^, Phe^27^, Val^36^, Val^38^, Ile^53^ and Ala^57^ (Figure 7c), most of which are highly conserved among HicA paralogues. Of the residues used in site-directed mutagenesis experiments, Gly^22^, Ser^23^ and H24A are located in the turn between the first and second β-strands. Not all the Gly^22^ and Ser^23^ resonances are visible in the spectra indicative of increased local dynamics in this short turn. Pro^41^ is located in the nearby long unstructured β3-α2 loop. Gly^14^, by contrast, is positioned at the other end of the molecule near the start of the β1 strand. The surface of the HicA protein is highly positively charged (Figure 7d).

The structure we report in the present study provides insights into the structural or catalytic roles of amino acids which are conserved across the HicA family of proteins. The β1-β2 and β3-α2 loops contain several conserved residues (Gly^22^, Ser^23^ and His^24^ in β1-β2 and Pro^39^, Pro^41^, Asp^44^ and Gly^48^ in β3-α2), suggesting that it is primarily this end of the molecule which is involved in catalysis and binding of the substrate and antitoxin. His^24^ and Gly^22^ probably have important catalytic and structural roles in the HicA–substrate complex. Surprisingly, the S23A mutation did not modify HicA function, despite the high degree of sequence conservation at this site and its proximity to Gly^22^ and His^24^. Several other conserved residues are likely to have a structural role by contributing to the hydrophobic core of the protein (Leu^10^, Trp^15^, Ile^53^ and Ala^57^). Gly^14^ is conserved, but our mutational studies show that it appears not to have a functional role. However, it occupies an unusual area of the Ramachandran plot, which although is accessible to other residues may be thermodynamically more stable for glycine residues. Roles for Lys^55^ and Gly^58^ remain unclear. Further (possibly double) mutations or HicA–HicB and HicA–substrate complex structures may be required to elucidate their function and confirm the active site.

At least eight known toxins exhibit RNase activity, although their structures, specificities and mechanisms vary [23,50]. For example, Zhu et al. [51] have reported that different MazF homologues from *M. tuberculosis* recognize different RNA sequence pentads, whereas RelE toxins preferentially cut codons between the second and third position of the binding site and upstream of a purine [52]. A previous study reported that RNase activity was associated with *E. coli* HicA [27]. However, it was unclear whether this was a result of a direct interaction between HicA and target RNA. We have shown His^24^ to be critical to the function of HicA which is consistent with RNase activity (the Catalytic Site Atlas [53] shows all known RNase active sites to contain at least one histidine residue). Further support for RNase activity comes from a model in which RNA was docked on to the PI-PfuI structure showing the RNA-binding site extending from a large endonuclease domain on to the middle domain where binding occurs across the β3-α2 loop and the top of the β-sheet [54]. Investigation of the charge distribution on the surface of H24A HicA shows the protein to be highly positively charged, especially across the β3-α2 and β1-β2 loops and the back of the β-sheet. A comparison of the structure and highly conserved residues does not suggest the obvious involvement of other histidine, lysine, glutamic acid or arginine residues in the possible RNase activity of His^24^. However, our HicA does contain a further histidine at residue 26 and many others contain one at residue 40 which is close in space to His^24^.

The closest structural homologues to HicA(H24A) are members of the YcfA/nrd intein domain superfamily: the middle (or stirrup) domain of PI-PfuI (PDB code 1DQ3) [55], the hypothetical protein TTHA1913 from *T. thermophilus* (PDB code 1WHZ), and the hypothetical protein YkfF from *E. coli* (PDB code 2HJJ). The structure is most closely related to that of TTHA1913 from *T. thermophilus* (Figure 7e), belonging to the YcfA-like family of protein structures in the SCOP classification [56]. Since TTHA1913 could be expressed and purified from *E. coli* for structure determination, whereas the expression of the *B. pseudomallei* HicA protein in *E. coli* was toxic [21,27], it seems likely that TTHA1913 is a non-toxic HicA homologue. However, comparison of the sequences and structures do not provide a clear indication of why TTHA1913 is not toxic to *E. coli.* Major structural differences lie between the unstructured β3-α2 loop (although sequence differences tend to be conservative) and the C-terminus; TTHA1913 has an additional C-terminal helix that is not present in HicA. The location of this helix, however, is near a negatively charged patch on the surface of the protein and distant from the β3-α2 and β1-β2 loops thought to be important in substrate binding.

In summary, we have demonstrated a role for the HicAB system in bacterial persistence and have demonstrated that bacterial persistence towards two different antibiotics is differentially dependent on HicA. This important finding provides a new insight into the phenomenon of multidrug resistance in persister cells. It is likely that this is only one of many mechanisms that regulate persister cell formation in *B. pseudomallei*. Finally, the structure we have obtained is the first of a HicA family protein which is proven to have toxic activity and our work provides new insight into structure–function relationships in this class of toxins.

## Online data

Supplementary data
