# Supplementary material for: The HicA toxin from Burkholderia pseudomallei has a role in persister cell formation
Source: Biochem J. 2014 Mar 28;459(Pt 2):333–44. doi: 10.1042/BJ20140073 (PMC3969222; doi:10.1042/BJ20140073)
Supplement: Supplementary data [file bj4590333add.pdf]

BJ Disease

Biochem. J. (2014) **459**, 333–344 (Printed in Great Britain) doi:10.1042/BJ20140073

online data

SUPPLEMENTARY ONLINE DATA
The HicA toxin from *Burkholderia pseudomallei* has a role in persister cell formation

Aaron BUTT*, Victoria A. HIGMAN†, Christopher WILLIAMS†, Matthew P. CRUMP†, Claudia M. HEMSLEY*, Nicholas HARMER* and Richard W. TITBALL*[1]

*Biosciences, College of Life and Environmental Sciences, University of Exeter, Geoffrey Pope Building, Stocker Road, Exeter EX4 4QD, U.K.
†School of Chemistry, University of Bristol, Cantock's Close, Bristol BS8 1TS, U.K.

Table S1 NMR assignment, structure calculation and validation statistics

Parameter	Values
Degree of assignment*	
Backbone (Cα, C', N and H^N) (%)	91.2
Side-chain H (%)	79.4
Side-chain non-H (%)	39.7
Number of restraints	
Distance restraints	
Intra-residue ($\|i-j\|=0$)	515
Sequential ($\|i-j\|=1$)	216
Medium range ($2\leqslant\|i-j\|<5$)	109
Long range ($\|i-j\|\geqslant5$)	172
Ambiguous	59
Total	1012
Dihedral angle restraints (Φ/Ψ)	94 (47/47)
Restraint statistics†	
RMSD of distance violations (Å)	0.019 ± 0.003
RMSD of dihedral violations (°)	0.27
Violations > 0.5 Å	0
Violations > 0.3 Å	0
Violations > 0.1 Å	9.8 ± 2.8
RMSD from idealized covalent geometry†	
Bonds (Å)	0.0031 ± 0.00017
Angles (°)	0.47 ± 0.0013
Impropers (°)	1.33 ± 4.97
Structural quality	
Ramachandran statistics*‡§	
Most favoured regions (%)	84.0 91.2
Allowed regions (%)	14.2/7.0
Generously allowed regions (%)	1.9/1.8
Disallowed regions (%)	0.0/0.0
CING ROG scores (R/O/G)* [1] (%)	20/22/58
Verify3D *Z*-score [2]	−2.57
Prosa II *Z*-score [3]	−0.74
Procheck *Z*-score (Φ/Ψ)‡ [4]	−1.14
Procheck *Z*-score (Φ/Ψ)‡ [4]	−1.71
MolProbity *Z*-score [5]	−1.57
Number of close contacts	2
Co-ordinates precision (RMSD)*§‖	
All backbone atoms (Å)	3.2/1.2/0.6
All heavy atoms (Å)	3.5/1.6/1.4

*Residues 5–59.
†Values reported by ARIA 2.3 [6].
‡Values reported by Procheck [4].
§Ordered residues (5–21, 23–32, 35–39 and 45–58) as calculated by PSVS 1.4 [3].
‖Residues in secondary structure (6–12, 16–19, 24–28, 35–39 and 50–58) as determined by DSSP [7].

[1] To whom correspondence should be addressed (email r.w.titball@exeter.ac.uk).
The chemical shifts and structure reported for HicA toxin from *Burkholderia pseudomallei* will appear in the Biological Magnetic Resonance Data Bank (BMRB) under accession number 19464 and in the PDB under code 4C26 respectively.

© The Authors Journal compilation © 2014 Biochemical Society

Biochemical Journal www.biochemj.org

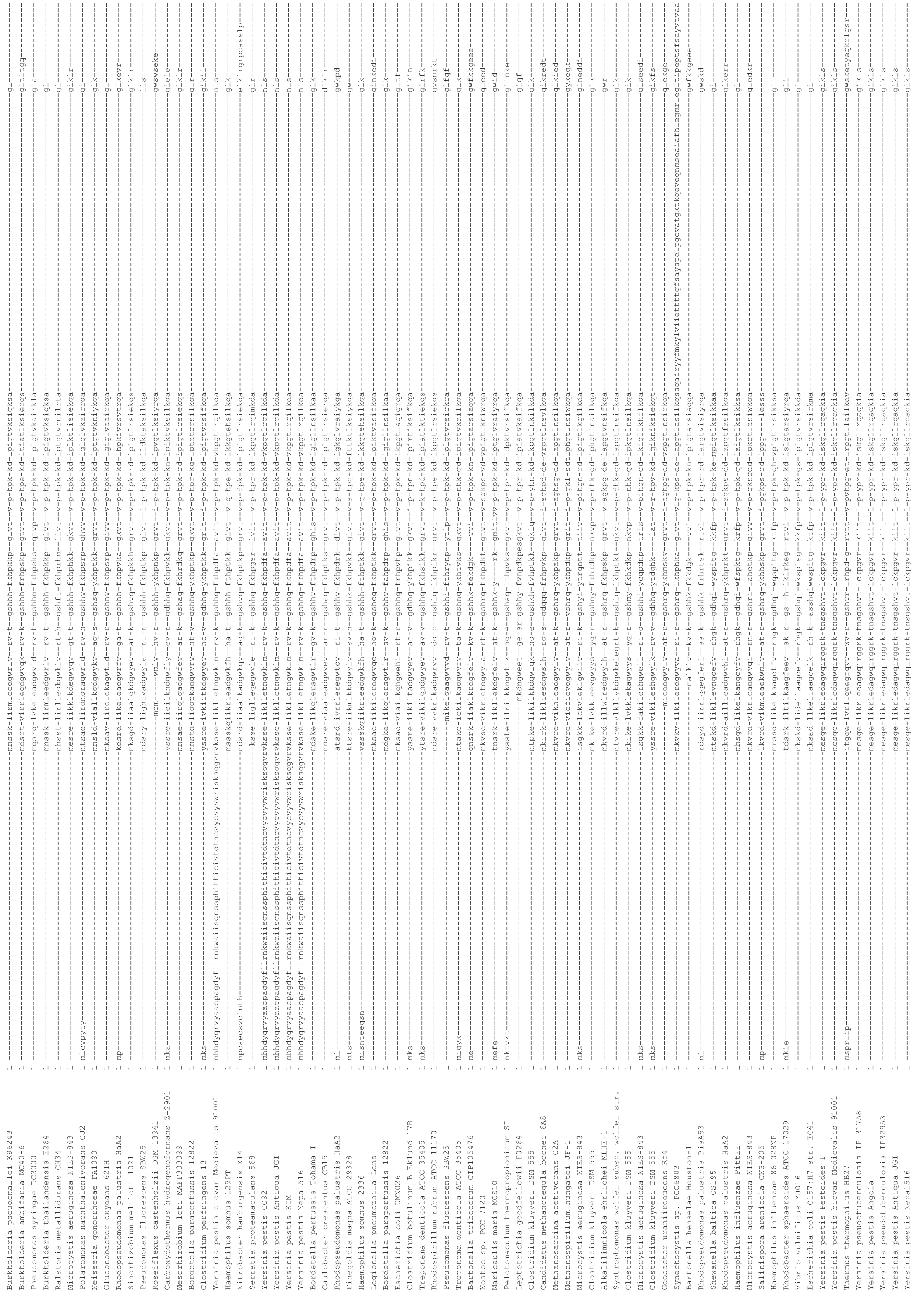

Figure S1 List of 75 HicA homologous sequences from differerent bacterial strains

These sequences were used for a subsequent ClustalW alignment and the most conserved residues are shown. A high-resolution version of Figure S1 is available to download (at http://www.biochemj.org/bj/459/bj4590333add.htm).

© The Authors Journal compilation © 2014 Biochemical Society

REFERENCES

1 Doreleijers, J. F., Sousa da Silva, A. W., Krieger, E., Nabuurs, S. B., Spronk, C. A., Stevens, T. J., Vranken, W. F., Vriend, G. and Vuister, G. W. (2012) CING: an integrated residue-based structure validation program suite. J. Biomol. NMR **54**, 267–283

2 Luthy, R., Bowie, J. U. and Eisenberg, D. (1992) Assessment of protein models with three-dimensional profiles. Nature **356**, 83–85

3 Bhattacharya, A., Tejero, R. and Montelione, G. T. (2007) Evaluating protein structures determined by structural genomics consortia. Proteins **66**, 778–795

4 Laskowski, R. A., MacArthur, M. W., Moss, D. S. and Thornton, J. M. (1993) PROCHECK: a program to check the stereochemical quality of protein structures. J. Appl. Cryst. **26**, 283–291

5 Lovell, S. C., Davis, I. W., Arendall, 3rd, W. B., de Bakker, P. I., Word, J. M., Prisant, M. G., Richardson, J. S. and Richardson, D. C. (2003) Structure validation by Cα geometry: ϕ, ψ and Cβ deviation. Proteins **50**, 437–450

6 Rieping, W., Habeck, M., Bardiaux, B., Bernard, A., Malliavin, T. E. and Nilges, M. (2007) ARIA2: automated NOE assignment and data integration in NMR structure calculation. Bioinformatics **23**, 381–382

7 Kabsch, W. and Sander, C. (1983) Dictionary of protein secondary structure: pattern recognition of hydrogen-bonded and geometrical features. Biopolymers **22**, 2577–2637

Received 20 January 2014; accepted 7 February 2014
Published as BJ Immediate Publication 7 February 2014, doi:10.1042/BJ20140073

© The Authors Journal compilation © 2014 Biochemical Society
